# The effectiveness of a value-based EMOtion-cognition-Focused educatIonal programme to reduce diabetes-related distress in Malay adults with Type 2 diabetes (VEMOFIT): study protocol for a cluster randomised controlled trial

**DOI:** 10.1186/s12902-017-0172-8

**Published:** 2017-04-04

**Authors:** Boon-How Chew, Rimke C. Vos, Sazlina Shariff Ghazali, Nurainul Hana Shamsuddin, Aaron Fernandez, Firdaus Mukhtar, Mastura Ismail, Azainorsuzila Mohd Ahad, Narayanan N. Sundram, Siti Zubaidah Mohd Ali, Guy E. H. M. Rutten

**Affiliations:** 1 Department of Family Medicine, Faculty of Medicine and Health Sciences, Universiti Putra Malaysia, 43400 Serdang, Selangor Malaysia; 2 Department of General Practice, Julius Center for Health Sciences and Primary Care, University Medical Center Utrecht, Huispost Str.6.131, P.O. Box 85500, 3508 GA Utrecht, The Netherlands; 3 Department of Psychiatry, Faculty of Medicine and Health Sciences, Universiti Putra Malaysia, 43400 Serdang, Selangor Malaysia; 4 Health Clinic Seremban 2, Jalan S2, A2, Seremban 2, 27300 Seremban, Negeri Sembilan Malaysia; 5 Health Clinic Port Dickson, KM 1, Jalan Seremban-Port Dickson, 71000 Port Dickson, Negeri Sembilan Malaysia; 6 Health Clinic Nilai, 71800 Nilai, Negeri Sembilan Malaysia; 7 Health Clinic Seremban, Jalan Rasah, 70300 Seremban, Negeri Sembilan Malaysia

**Keywords:** Diabetes-related distress, Self-care, Depression, Diabetes education, Quality of life, Type 2 diabetes mellitus, HbA1c, Blood pressure, LDL-cholesterol

## Abstract

**Background:**

Type 2 diabetes mellitus (T2DM) patients experience many psychosocial problems related to their diabetes. These often lead to emotional disorders such as distress, stress, anxiety and depression, resulting in decreased self-care, quality of life and disease control. The purpose of the current study is to evaluate the effectiveness of a brief value-based emotion-focused educational programme in adults with T2DM on diabetes-related distress (DRD), depressive symptoms, illness perceptions, quality of life, diabetes self-efficacy, self-care and clinical outcomes.

**Methods:**

A cluster randomised controlled trial will be conducted in 10 public health clinics in Malaysia, all providing diabetes care according to national clinical practice guidelines. Patients’ inclusion criteria: Malay, ≥ 18 years with T2DM for at least 2 years, on regular follow-up with one of three biomarkers HbA1c, systolic blood pressure and LDL-cholesterol sub-optimally controlled, and with a mean 17-item Diabetes Distress Scale (DDS-17) score ≥ 3. The intervention consists of four sessions and one booster over a period of 4 months that provide information and skills to assist patients in having proper perceptions of their T2DM including an understanding of the treatment targets, understanding and managing their emotions and goal-setting. The comparator is an attention-control group with three meetings over a similar period. With an estimated intra-cluster correlation coefficient ρ of 0.015, a cluster size of 20 and 20% non-completion, the trial will need to enroll 198 patients. Primary outcome: the between groups difference in proportion of patients achieving a mean DDS-17 score < 3 (non-significant distress) at 6 months post-intervention. Secondary outcomes will be the differences in the above mentioned variables between groups.

**Discussion:**

We hypothesize that primary and secondary outcomes will improve significantly after the intervention compared to the comparator group. The results of this study can contribute to better care for T2DM patients with DRD.

**Trial registration:**

ClinicalTrials.gov NCT02730078. Registered on 29 March 2016, last updated on 4 January 2017.

**Electronic supplementary material:**

The online version of this article (doi:10.1186/s12902-017-0172-8) contains supplementary material, which is available to authorized users.

## Background

The prevalence of type 2 diabetes mellitus (T2DM) has increased during the past decades in many countries, with persistent high disease burden and healthcare cost [1]. In Malaysia, the prevalence of T2DM, diabetes-related complications and poor disease control and management are also increasing [2]. For many patients, it is difficult to cope with their chronic disease in daily life [3, 4]. As a result, many are experiencing complications (cardiovascular diseases, nephropathy, retinopathy and neuropathy) and psychological problems from uncontrolled diabetes [3, 5–7].

Studies in the United States (US) [8] and Germany [9] showed T2DM patients displaying one to twofold higher rates of affective disorders such as major depression, general anxiety, panic disorder and dysthymia compared to healthy adults, with the prevalence of depressive symptoms and distress 60–73% higher than the other affective and anxiety disorders [8]. We could demonstrate high rates of emotional burden experienced by patients with T2DM: about half of them had diabetes-related distress and about 30% had any degree of depression, with about 10% severe depression [10]. In an earlier study in 12 Malaysian public health clinics using the Depression, Anxiety and Stress Scale (DASS) questionnaire, the prevalence of depression, anxiety and stress symptoms among T2DM patients were 11.5, 30.5 and 12.5% respectively [11]. In a US survey the prevalence of untreated serious psychological distress was higher in T2DM patients compared to the total population and the non-diabetes population, and associated with young age, low education levels, low household income, obesity, current smoking, no leisure-time physical activity and presence of one or more micro- or macro-vascular complications [12]. Newly diagnosed T2DM patients who experience psychosocial problems often use negative coping strategies and expect more frequently that diabetes would negatively affect their future compared to patients who did not experience psychosocial problems [13]. Patients treated with insulin experienced higher diabetes distress compared to tablet- or diet-treated patients, and this emotional burden might cause insulin non-adherence if it is not improved [14, 15].

Diabetes-related distress (DRD) or diabetes distress is defined as the patient’s concerns about his/her disease management, (social) support, emotional burden, and access to care [16]. DRD and stress in a general sense were perceived similar in earlier literature until a more specific measure for DRD was developed [17]. Fisher et al. reported that what has been widely defined as “depression” among T2DM patients may be either a major depressive disorder or/and DRD, with only the latter displaying significant time-concordant relationships with glycaemic control (HbA1c) [18]. It is likely that DRD and depression are on the same scale of emotional disorders, but differ in severity, with other emotional disorders such as stress, dysthymia, anxiety in between [19]. Confusion between DRD and depression has recently been addressed and the authors had consented on emotional distress as the term to include both depression and DRD [20]. Since T2DM patients with DRD might not be capable of adequate self-care [21] it is recommended to take DRD into account in diabetes care even when it is considered mild [20].

Psychological interventions to improve both DRD and glycaemic control vary widely in their content and effectiveness [22, 23]. Interventions that addressed emotional aspects of coping with diabetes and took into account DRD showed promising results [24]. Recent systematic reviews show that psycho-education consisting of both emotion and cognition components are likely to be effective in reducing DRD [25, 26]. However, providing any psychological/emotional support by the healthcare team might pose a challenge to the existing healthcare system [20, 27]. Interventions with a highly specialised content require complex skills, involve physicians and/or psychologists and may require many sessions over a long period. Such interventions are hard to implement at the primary care level with its usually high patient-load, relatively low use of technologies, under-trained staff and budget constraints [28]. Effective psychological interventions in the primary care setting could be group-based [29], individualised to a person’s lifestyle, respecting an individual’s habits and routines; and can be conducted by health care providers [23, 26, 29–31] as well as non-expert mental health coaches [32].

### Conceptual framework

**Fig. 1:**
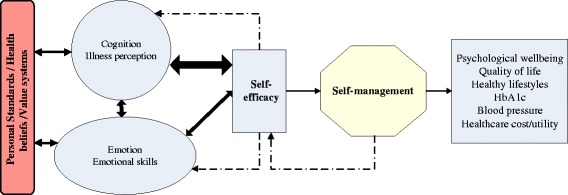
Conceptual framework

Optimal diabetes care requires emotionally healthy as well as cognitively competent patients [33, 34]. Emotion-focused and cognition-focused coping approaches have been shown to affect differentially on changes in lifestyle leading to psychological and physical benefits in adult T2DM patients [35]. Hence, both emotion and cognition are psychological aspects that should be taken in combination to achieve effective self-management [36]. It is hypothesized that emotional and cognitive domains that are in harmony with the personal value system and purpose in life may improve self-efficacy in patients with T2DM (Fig. 1) [37–41]. The Social Cognitive Theory of Self-regulation [42, 43] focuses attention on personal health beliefs and value systems as well as addresses emotional skills, with appropriate provision for the cognitive understanding of disease [44]. Patients’ negative beliefs about illness could influence physical and mental functioning [45, 46], and cause DRD and depression [47]. Furthermore, partners’ illness perception of T2DM could also affect self-management behaviours and psychological wellbeing of the patients [48–50]. Therefore, realistic illness perception in both the patients and their significant others will increase self-efficacy that would lead to better self-care and disease control.

The emotional consequences of diabetes and its management are not limited to patients alone [51]. Social control attempts may have dual effects (appreciation and hostility) on recipients’ well-being, such that improved health behaviours may occur at the cost of increased emotional distress [52]. Therefore, spouses and family members can impede or facilitate the patient’s self-management [53, 54]. In this trial we will address the behaviours, feelings, and thoughts of both patients and their partners/significant others, and involving them throughout the programme’s activities for the patients. Since backsliding and regression to previous unhealthy lifestyles are not unusual for many T2DM patients, regular reinforcement by their significant family members of the uphold values, emotional skills and knowledge is necessary for the sustainability of the desired perceptions and behaviours [54, 55].

Accordingly, we developed an intervention that takes the patients’ beliefs into account besides addressing both the emotional and cognitional needs, involving their spouses or family members. The intervention will be delivered by nurses at the primary care level, targeting essential domains of health behaviours. It aims to provide information and skills to assist patients in adequate judgement and perceptions of their T2DM, including an understanding of the treatment targets, understanding and managing their emotions and goal-setting [56]. The intervention is culturally appropriate and competent through its targeted participants of single ethnicity and delivered by healthcare providers of the same ethnicity who will be trained to deliver the intervention in a compassionate and sensitive manner, taking into account the patients’ cultural beliefs, behaviours and needs [57].

### Aims of the trial

This study aims to assess the effectiveness and appreciation of a value-based emotion-cognition-focused educational programme in Malay adults with T2DM (VEMOFIT) delivered by Malay health clinic nurses. As the nurses are the deliverers of the intervention, the health clinics are the unit of randomisation. A cluster randomisation can prevent contamination through social and professional interactions between patients-patients and patients-nurses, respectively, and by preference of patient or physician.

It is hypothesized that the VEMOFIT will be appreciated by the participating patients and will be significant in reducing DRD, depressive symptoms, improving illness perception, health related quality of life, self-efficacy, health behaviour change (self-management), disease control (HbA1c, blood pressure, lipids) and healthcare utilization compared to before the intervention, and compared to attention meetings plus usual care. The rationale for using an attention control group (instead of a ‘usual care’ control group) is to distinguish the effectiveness of an intervention from personal attention provided by the healthcare team. Participants in both groups receive personal attention, but only the intervention group receives the VEMOFIT. The control group in this study gets significantly more attention with regards to their healthcare experience compared to usual diabetes care.

## Methods/Design

### Study design

The trial incorporates an active intervention over four sessions in six weeks, one booster session three months later and two follow-up evaluations. We position this trial towards the explanatory end of the explanatory-pragmatic continuum in Malay T2DM patients and the use of attention control as the comparator instead of usual care. We have written trainer’s manuals and training sessions for the trainers in both the VEMOFIT and the attention control groups. The trial is reported according to the Standard Protocol Items: Recommendations for Intervention Trials (SPIRIT) [58] and will be reported in accordance to the Consolidated Standards of Reporting Trials (CONSORT) Statements: extension to cluster randomised trials and non-pharmacologic treatment [59].

### Study setting - the clusters

Clusters are the public health clinics (HC) with more than 1000 T2DM patients in urban and suburban areas in the state of Negeri Sembilan, Malaysia. These clinics are the bigger public HCs with resident doctors and family medicine specialists (FMS) providing diabetes care in accordance to the Malaysian national clinical practice guideline [60]. Other inclusion criteria: the HCs have at least two nurses available to be trained for the intervention and the HC will/should not be participating in other similar studies. Out of 14 public HCs that fulfilled the above criteria and were invited to participate, 10 agreed.

These HCs are generally equipped with in-house facilities ranging from medical laboratory tests, plain X-rays to pharmacy. The public primary care service usually comprises of a multi-disciplinary team approach in patient care consisting of nutritionists or dieticians, pharmacists, physiotherapists, occupational therapists and paramedics who have undergone specialised training to provide services such as diabetes education. Patients in these HCs generally are managed by medical and health officers (M&HO) and supported by specialised nurses and dieticians/nutritionists. Nurses contribute in measuring clinical parameters during patients’ visits and give health education at the appointment day. Under the current health system, every patient diagnosed with T2DM will receive a green booklet which will be kept by the patient. This is accompanied by a bigger green medical record book (kept at the health clinics) that records all information pertaining to the care provided. Patients who have complications are referred to the hospitals either for admission or for shared care as outpatients with the hospitals’ specialists including endocrinologists or diabetologists.

### Participants

Patients will be screened using a structured case report form for eligibility. Eligible patients are Malay patients aged ≥ 18 years old with T2DM for at least 2 years. Their records must show that they are on regular follow-up with at least three visits in the past year and with blood results from the past 3 months. We will exclude patients who are enrolled in other clinical studies, pregnant or lactating, with known psychiatric/psychological disorders that could impair judgments and memory and patients who cannot read or understand English or Malay. Clinic staff involved in the screening will be instructed not to exclude any patients unless they fulfil one of the exclusion criteria, to avoid selection bias. In addition, participating patients must have current DRD with a mean 17-item Diabetes Distress Scale (DDS-17) score ≥ 3 and showing poor disease control, i.e. having one of these three biomarkers: HbA1c ≥ 8%, blood pressure ≥ 140/90 mmHg and LDL-C ≥ 2.6 mmol/L. Patients who scored ≥ 20 on the 9-item Patient Health Questionnaire (PHQ-9) which suggests a severe depression will be referred for further psychological attention and will not be invited to participate.

### Recruitment

Eligible patients are recruited consecutively from the 10 participating HCs during their routine scheduled visits. This screening phase (T0) will provide the baseline characteristics, including DRD and depressive symptoms for the decision of inclusion into the study. We use a modified informed consent procedure to ensure that patients are unaware of the two different intervention programmes.

After randomisation, participants will be informed about the programme and the first meeting by the clinic nurses. During this first meeting, which is not part of the intervention, baseline measurements will be performed (see further below), and participants’ eligibility will be further confirmed. All research materials that contain personal information such as the questionnaires will be coded to safe-guard confidentiality of the participants throughout the study.

### Cluster randomisation

**Fig. 2:**
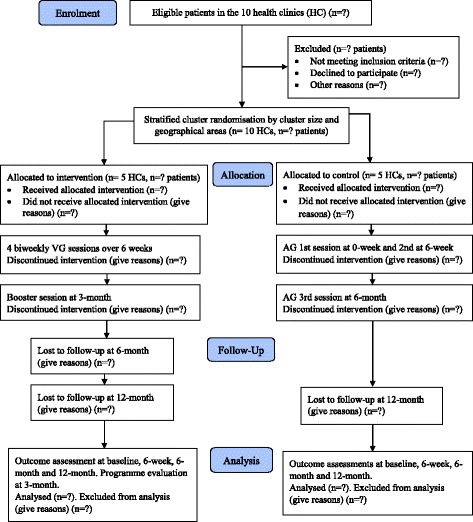
Flow of health clinics and participants through trial. VG: VEMOFIT group; AG: attention-control group

In assigning the HCs to the VEMOFIT group (VG) or attention-control groups (AG), randomisation will be carried out after stratification by cluster size and geographical areas of the 10 HCs. A member of the Data Management Services team at the University Medical Center, Utrecht will carry out the randomisation. Figure 2 shows the flow of HCs and participants through trial.

### Intervention

VEMOFIT is based on the above conceptual framework and developed with the Medical Research Council Framework for the evaluation of complex interventions to improve health [61]. The content of VEMOFIT was developed in The Netherlands by a team of experts consisting of a psychologist, a primary care diabetologist, a FMS and epidemiologists with experience in behavioural interventions for people with chronic diseases. We evaluated its content validity with Malaysian psychologists, FMSs and nurses. Its curriculum will be printed as a training manual for the nurses and as a Workbook for the participants. The intervention will be delivered by trained nurses (for training: see further). A process evaluation will be conducted (see further for details) to ascertain that the intervention is generally delivered as intended. Similarly, the attention-control programme (see further) has been developed and validated by the same teams. Its content will be provided to the nurses as a training manual and guidebook.

#### A pilot study

During a pilot study, the acceptance of the VEMOFIT content, face validity and feasibility will be tested. This will be arranged after the training workshops for the nurse-coaches, separately for the VEMOFIT and the attention-control groups. Real patients with similar inclusion and exclusion criteria will be recruited from a non-participating health clinic in Negeri Sembilan and invited to attend the interventional programmes delivered by trained nurse-coaches. All nurse-coaches will have an opportunity to deliver at least one structured session, all will be given feedback on their own and peers’ performances. This pilot study had been completed, and its outcomes had informed and were incorporated into this study protocol.

#### The VEMOFIT intervention

**Fig. 3:**
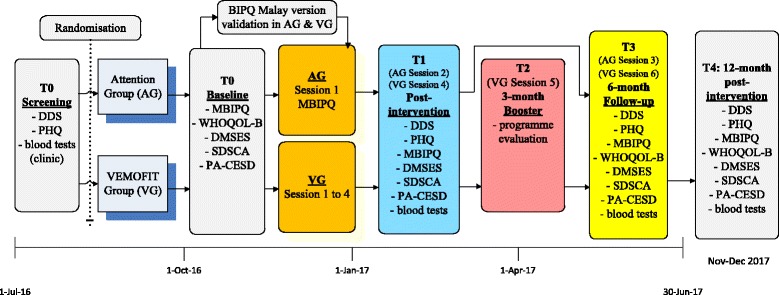
Timeline of the interventions. DDS: 17-item Diabetes Distress Scale; PHQ: 9-item Patient Health Questionnaire; MBIPQ: Malay version Brief Illness Perception Questionnaire; WHOQOL-B: World Health Organization Quality of Life- Brief; DMSES: Diabetes Management Self Efficacy Scale; SDSCA: Diabetes Self-Care Activities; PA-CESD: Positive Affects subscale of the Center for Epidemiologic Studies Depression Scale

The VEMOFIT intervention involves four biweekly 2 hours sessions over a period of about six weeks, and a booster at 3 months follow-up (Fig. 3 and Additional file 1). All the sessions will be in the patient’s own HC. The intervention is group-based and consists of a mixture of 1) exploring illness perceptions and personal meanings of diabetes, 2) cognition-focused education on diabetes and practical skills in self-management and 3) emotion-focused training on recognising emotions in the self and others. Each group will consist of 10 to 12 participants, with equal numbers of patients and their significant others. There will be two groups in each clinic in the VG in order to achieve the required sample size of about 100 (see below). The outline of the content of the intervention is listed below, a detailed description of the interventions will be recorded in the training protocols and in presentation slides.

The intervention will provide information, practice examples, exercises/self-tests and homework assignments in the first three meetings (start, week 2 and week 4). Patients receive feedback on their homework assignments from the trained nurse-coaches at the beginning of each session. During the intervention phase, the nurse-coaches will be supported and supervised by the research team. In the programmes, specific issues (e.g. oral medication or insulin, co-morbidities and complication types) may not apply to all participants, but can serve to illustrate the common features of living with diabetes and its emotional challenges, while respecting individual differences.

#### First meeting

During the first meeting, details are given about the time, locations, and duration of the meeting. Baseline questionnaires (except the DDS-17 and PHQ-9, as these data have been collected during screening for eligibility) will be filled in by all participants. Questionnaires are provided in English and Malay. Data collection will be carried out in the HCs by the health clinics’ nurses. Nurses who deliver the intervention will not collect data from the patients. The first intervention session will be conducted within 14 days after this meeting.

#### First session (week 0): illness perception, value exploration & T2DM disease education

During the first session, the focus is on illness perceptions. Patients will be guided in reflecting on their perceptions of diabetes mellitus and its effects, impact and meanings. The coach will discuss the participants’ perceptions on diabetes and assist them to crystalize their thoughts. Afterwards participating patients should write down their personal thoughts in the patient’s workbook. Patients and their significant others are encouraged to further consolidate these perceptions with each other during the session and in group discussion, and with other family members if needed at home and to share their thoughts on the second session. The second part of this session is on education of T2DM as a disease, screening needs, control targets, healthy life-styles and medication management. An M&HO from the health clinic will be trained to deliver this short lecture. The nurse-coaches will be present throughout this session and assist the patients in scoring their cardiovascular risk profile. At the end, the nurse-coaches will inform patients on the relevance of the second and third sessions in adjusting their illness perceptions from the perspectives of emotion and cognition. Patients will receive a set of leaflets with more information on T2DM [62], and a diary as homework to record their feelings, symptoms, diet, physical activity and visits to emergency departments or hospitalisation if any.

#### Second session (week 2): emotional skills- understanding emotions

The focus of the second session is on emotional skills training, in particular about understanding emotions. Emotional skills consist of understanding and managing emotions in oneself and in others and were modified from the Train the Trainer Workbook [63]. It begins with a discussion on the past two weeks experience as recorded in the diary, results from the family sharing of the personal meanings and values by the patients, and clarification of queries about T2DM. The M&HO who delivered the lecture in the first session will be present for this discussion. The nurse-coach gives a short talk on the nature of emotions, its vocabulary, somatic manifestation, sources and effects. Participants will be educated about the fact that every patient has a certain degree of distress from his/her T2DM. Unjustified illness perception and unattended emotions may cause DRD. Exercises on emotional skills in the workbook will involve both the patients and their significant others. At the end of this session, a relaxation technique is taught to and practiced by the participants. The patients are required to continue recording their emotions in the diary for the next two weeks. An information leaflet on providing social support is briefly explained and handed out to the participants’ significant others.

#### Third Session (week 4): emotional skills- managing emotions

The focus of the third session is on managing emotions. It starts with a discussion on the emotional experiences based on the diary, relating life experiences with personal value and experience in receiving and providing social supports. The nurse-coaches will deliver a short talk on emotions and T2DM, highlighting the causes and effects of emotions on diabetes control. Skills in managing emotions are taught and practiced through some exercises. Preventative strategies will modify the type and magnitude of emotions that are going to be experienced in an anticipated situation or event; responsive strategies will enhance, reduce or curtail an emotional experience after it occurs. This session ends with a practice on the relaxation technique.

#### Fourth session (week 6): value-cognition-emotion combined

The fourth session starts with a discussion on the diary with an emphasis on the emotions experienced, their possible modification and the roles of personal values. A short summary of the VEMOFIT by the nurse is followed by a discussion to further consolidate lessons and skills learned in the past three sessions. This leads to the focus of the last session of the intervention, using the gained emotional skills and knowledge to set short- and long-term goals. The nurse-coaches will coach and help the patients to formulate plans to reach these individualised goals. At the end of the session, participants will be evaluated.

#### Booster session (week 18)

The booster session has an identical content as the fourth session. The goals formulated during the fourth session are evaluated. The barriers and facilitators to self-management and goals are discussed in relation to the experienced emotions, illness perception, and knowledge gaps. If necessary, goals are revised and altered according to needs. The nurse-coach will provide a short summary of the key elements of the programme again. Participants will be encouraged to attend the final meeting.

#### Six months (week 30) and twelve months meeting (week 54)

During this meeting participants will fill in questionnaires again. This will be followed by an interactive discussion on self-management goals, the barriers and the facilitators, and reflection on the emotional skills/experience.

#### Nurses’ training

Nurses from the participating HCs are invited to participate in a 2-days training course. The course will be provided by the investigators and will include practical coaching skills in encouraging reflection, communication skills, emotional skills, emotion diary documentation and adult learning theory [64–67]. In addition, the nurses will be taught on questioning about illness perceptions to encourage reflection, strengthen self-efficacy and to intervene on emotional conflicts; general information about diabetes management in the primary care setting; and discuss the types of anti-diabetic medication and their proper use. Lastly, the principal investigator and project manager will also discuss the different aspects of the study.

To check whether the nurses perform the programme correctly and use the information from the training, we will evaluate the actual performance of the trained nurses in conducting all four VEMOFIT sessions in a group of patients and their significant others from another non-participating HC in the same state of Negeri Sembilan. The trained nurses will complete self-assessments of each session on aspects of the group session, including overall success, coverage of all learning topics, patient participation, responsiveness to patient concerns, and comfort level in facilitating the group.

### Attention-control health clinics

Patients in the HCs randomised to the AG, will receive the usual diabetes care. At three moments patients (not their significant others) will be gathered in groups of 10–12 people to give them attention, but not the theoretical support of the programme. These sessions will include general discussion on feelings about and coping with T2DM (session 1), social support at home and satisfaction with treatment (session 2) and care received at the respective clinics (session 3). The first session will be given at the same time as the first session in the VG, the second one concurrent with the fourth VG intervention session and the third meeting will held 30 weeks after the start of the trial. Patients will receive the same set of educational leaflets on diabetes. One nurse from each of the HCs in the AG will be trained for one day to provide the “attention” and lead the discussion. They will receive a manual.

It will be clearly stated during the course of the study that all participants are allowed to make use of additional mental health care services if they feel a need to do so. The timetable for the complete intervention and booster sessions is shown in the Additional file 1.

### Participant programme evaluation

We will evaluate patients’ satisfaction with VEMOFIT at the end of the booster session with a self-developed 12-item 5-point Likert-type self-report survey. We will investigate the extent to which the intervention helped participants improve their illness perceptions, emotional skills and to meet diabetes self-management goals; whether group sessions were scheduled to convenience; whether setting and nurse-coach were helpful, whether the programme was delivered in a culturally sensitive and understandable context. Finally, participants will be asked to comment on aspects of the intervention that they liked/disliked the most.

### Outcomes

#### Primary outcome

Diabetes-related distress, measured with the 17-item Diabetes Distress Scale (DDS-17)

#### Secondary outcomes

1. Depression, measured with the Patient Health Questionnaire (PHQ-9)
2. Illness perception measured with the Brief Illness Perception Questionnaire (BIPQ)
3. Quality of life, measured with the World Health Organization Quality of Life- Brief (WHOQOL-BREF) questionnaire
4. Self-efficacy, measured by the Diabetes Management Self Efficacy Scale (DMSES)
5. Self-care behaviours, measured with the Diabetes Self-Care Activities (SDSCA) scale
6. Positive emotions measure by the Positive Affects subscale of the Center for Epidemiologic Studies Depression Scale (PA-CESD)
7. Cardiometabolic status: HbA1c, blood pressure, lipid profiles
8. Health-care utilization and hospitalisation

We will measure effects of the VEMOFIT on patient’s level by self-reported outcomes as listed in Table 1. In addition, we will use a case record form to retrieve data on co-morbidity, diabetes-related complications, duration of diabetes, glycaemic control (HbA1c), blood pressure control, lipids control, and number and type of medication use. Nurse-coaches will not be taking the blood pressure measurement. A questionnaire will capture demography data such as age, gender, ethnicity, religiosity, marital status and educational level. Health care utilization comprises the number of visits to the health clinic/hospitals/referrals to other health care professionals over the past year. This will be collected prospectively during the study by means of a questionnaire and a diary.

**Table 1:** Description of questionnaires

Questionnaire	Description	Score range
17-item Diabetes Distress Scale (DDS-17) [16]	Assesses problems and hassles concerning diabetes during the past 1 month. Four sub-scales: emotional burden (EB), physician-related distress (PD), regimen-related distress (RD) and diabetes-related interpersonal distress (ID).	Likert scale scores from 1 (not a problem) to 6 (a very serious problem). Total scale score plus 4 sub scale scores. A mean item score of ≥ 3 (severe distress) is considered a level of distress worthy of clinical attention [74].
The Brief Illness Perception Questionnaire (BIPQ) [75]	The scale has nine items providing simple and rapid assessment of illness perceptions. It measures patients’ cognitive and emotional representations of their illness including consequences, timeline, personal control, treatment control, identity, coherence, concern, emotional response, and causes.	Eight items are rated using a 0-to-10 response scale except the item 9 on causal question. For example when measuring understanding about the illness, the scale ranges from 0 (don’t understand at all) to 10 (understand very clearly). The causal open-ended response item asks patients to list the three most important causal factors for their diabetes.
Patient Health Questionnaire (PHQ-9) [76, 77]	Nine items refer to symptoms experienced by patients during the 2 weeks prior to answering the questionnaire in making diagnosis and assessing severity of depression.	Scores range from 0 to 27, as each of the nine items is scored from 0 (not at all) to 3 (nearly every day). PHQ-9 scores of 5, 10, 15, and 20 represents mild, moderate, moderately severe, and severe depression, respectively.
World Health Organization Quality of Life- Brief (WHOQOL-BREF) [78, 79]	Twenty-four items assessing health-related quality of life in the past four weeks. It produces four quality of life domains and scores, 1) Physical domain, 2) Psychological domain, 3) Social Relationships domain and 4) Environment domain. Two extra items examine separately: question 1 asks about an individual’s overall perception of quality of life and question 2 asks about an individual’s overall perception of his or her health.	Items have Likert scale from 1 to 5. Higher scores denote higher quality of life. The mean score of items within each domain is used to calculate the domain score. Mean scores are then multiplied by 4 in order to make domain raw scores comparable with the scores used in the WHOQOL-100.
Diabetes Management Self Efficacy Scale (DMSES) [80–82]	Twenty items, to evaluate patients’ confidence in managing their disease in terms of blood glucose, diet, and exercise.	Scores range from 10 if the respondents “certainly can do” to 0 if they “cannot do at all.” Total scores range from 0 to 200.
Summary of Diabetes Self-Care Activities (SDSCA) [81, 83, 84]	Eleven items that measure patients’ daily activities during the past 7 days in relation to diet, exercise, blood sugar, foot care and smoking behaviour.	Ten items are rated on an 8-point Likert scale, measuring how many days an activity is performed in the last week. One item measures smoking status (yes/no) and the amount of cigarettes smoked in the last week. Each of the domains is measured separately.
Positive affects subscale of the Center for Epidemiologic Studies Depression Scale (PA-CESD) [85]	Four items on positive affects such as self-esteem, hopeful, happy and enjoying life.	Items are assessed for the past 1 week on a scale of four possible responses; 0) less than 1 day, 1) 1–2 days, 2) 3–4 days; and 3) 5–7 days. Higher scores indicate more positive feelings

### Definitions

T2DM is defined present if the case record fulfils all these criteria: (i) either documented diagnosis of T2DM according to the established criteria [68, 69] or (ii) current treatment with life-style modification, oral anti-hyperglycaemic agents or insulin. Hypertension is diagnosed if a patient is being treated with blood pressure lowering agents and regarded as ‘controlled’ if the most recent blood pressure (BP) is < 140/90 mmHg. Hyperlipidaemia is considered to be present if a patient is on statin or fibrate treatment or has a low density lipoprotein-cholesterol (LDL-C) ≥ 2.6 mmol/L, triglyceride (TG) ≥ 1.7 mmol/L or high density lipoprotein-cholesterol (HDL-C) ≤ 1.2 mmol/L. A LDL-C < 2.6 mmol/L and HbA1c < 7.0% are regarded as treatment targets [60, 69]. Diabetes-related complications (retinopathy, nephropathy and diabetic foot problems (DFP), ischemic heart disease and stroke) are retrieved from the patients’ medical records. Nephropathy is diagnosed if on at least two occasions any of the following has been recorded: microalbuminuria, proteinuria, serum creatinine > 150 mmol/L or estimated glomerular filtration rate < 60mls/min (calculated using Cockroft-Gault formula). DFP comprises foot deformity, current ulcer, amputation, peripheral neuropathy or peripheral vascular disease.

### Sample size

In a previous study [23] the DDS-scores were normally distributed with a standard deviation of 0.8. If the true difference between the experimental and control means is 0.4 [23], we will need to study 64 experimental subjects and 64 control subjects with a power of 0.8 and a Type I error of 0.05. In a recent local study in 10 health clinics, the intraclass correlation coefficient (ICC) for HbA1c was 0.011 [70]. For cluster randomised clinical trials the standard sample size calculation needs to be inflated by a factor: 1 + (n – 1) ρ, where n is 20 (the average cluster size) and ρ is 0.015 (the estimated ICC for this study) [71]. This inflation factor is 1.285. This gives a total sample size of 165. Since we anticipate a 20% drop-out rate, we will ask eligible patients for participation until at least 198 have signed informed consent.

### Statistical analyses

Data will be entered and checked for accuracy by two separate person before analysis. The principal investigator has the overall responsibility for compilation, maintenance and management of the study database. The database is stored on a password-protected computer in a locked office. Analyses will be carried out by both an intention-to-treat approach and a per protocol analysis. Data will be checked for normality and multicollinearity, and if necessary transformed. Estimates will be obtained with PASW 22.0 (SPSS, Chicago, IL).

One-way analysis of variance (ANOVA) and *χ* ^2^ tests, as appropriate, will be conducted to test for baseline differences across the two treatment conditions and to examine differences in outcomes between dropouts and continuing participants. The difference between the groups will be analysed using a 3-level mixed model to account for clustering of measurements within patients and patients within general practices. The random part of the model will include a random intercept per practice and an unstructured correlation matrix for the correlation of measurements within patients. The fixed part of the model will include the variables time (categorical), treatment group, a group*time interaction and the baseline DDS-17 score; the difference in DDS-17 score at 6-month will be tested using a linear contrast. If necessary a multiple imputation technique will be used for missing data. A calculated 95% confidence interval and two-sided α of 0.05 will be used to test significance.

We will evaluate how baseline predictors are related to baseline DDS-17 score (cross-sectional analyses). Second, we examine how the baseline predictors are related to linear change in DDS-17 score over time (prospective analyses). Third, we will explore a set of time-varying covariates: how changes in a predictor over time are related to changes in DDS-17 score over time. Similar statistical modelling will be carried out on secondary outcome measures. Continuous outcome variables will be analysed using linear mixed models and binary outcome variables using generalized linear mixed models.

#### Treatment fidelity

To monitor and enhance the reliability and validity of the behavioural interventions [72], we will use elements of a framework developed by Bellg et al. to make treatment fidelity explicit [73]. The framework consists of five strategies, as described in Table 2.

**Table 2:** Framework of treatment fidelity strategies

Treatment design	Information provided about intervention • Length of intervention programme sessions (2 to 2.5 h) • Number of intervention programme sessions (5) • Duration of intervention (5 months) Information provided on standardized care • Number of routine visits to HCs (every 2 to 4 months)
Training procedures	Training of nurses outlined • A one-day training session • One day pilot test with real patients (4 sessions) with feedback from investigators (2 sessions) • A one-day refresher session
Delivery of treatment	Assurance that intervention has been delivered as per protocol • Trainer’s manual • Patient’s workbook • Principle investigator contacts every nurse-coach after each of their sessions • Nurse-coaches fill in log diaries • Focus groups with participants and nurse-coaches
Receipt of treatment	• Record of each participant’s attendance at meetings • Nurse-coach takes a record at each meeting

#### Process evaluation

Data from the nurse-coaches log diaries and the project manager’s record of contact with the nurse-coaches is analysed, in addition to the below qualitative data, to evaluate the process of the implementation of the intervention.

#### Qualitative evaluation

A qualitative analysis will be carried among patients and nurse-coaches to evaluate their attitudes to the VEMOFIT programme and their experience of its delivery in intervention HCs. Semi-structured interviews and focus group discussion will be held among a purposive sample of at least 4 nurses and 25 patients, respectively, to explore questions such as:

- Is VEMOFIT an acceptable intervention?
- How does VEMOFIT work?
- Was the intervention successfully implemented?
- Did the programme benefit the nurse-coaches?
- Will people take up this service in the future if it was offered?
- Did the intervention and standard diabetes care significantly increase the practice staff’s workload?

The interviews will be audio-taped and transcribed verbatim. Several methods of improving validity of qualitative research will be conducted including respondent validation.

## Discussion

Since psychological problems and poor illness perception are already prevalent in the early stages of T2DM [10] and early adequate diabetes control is desirable, the primary care setting seems the appropriate clinical setting for this study. DRD is an important outcome in this respect. Emotion-cognition focused psychological intervention is most promising in improving DRD in T2DM patients and at primary healthcare setting [26]. Hence, we think the VEMOFIT intervention could be considered as a minimal psychological intervention needed to improve DRD in adults with T2DM in Malaysian public health clinics.

This trial is positioned towards the explanatory end of the explanatory-pragmatic continuum. We would like to maximize its external validity by having few exclusion criteria and by allowing flexibility in the delivery style of the intervention by the nurse-coach and in medical management decisions by the treating physician at the respective HCs. Internal validity is maximized by decreasing contamination bias through cluster randomisation, decreasing observer and assessment bias through baseline data collection prior to randomisation, treatment fidelity monitoring, process evaluation and blinding the data analysis.

With the results of this study, primary care professionals and practice will be better informed and prepared to provide psychological support to these patients.

## Additional file

Additional file 1: The timetable. T2DM: type 2 diabetes mellitus; VG: VEMOFIT group; AG: attention-control group. VEMOFIT: Value-based EMOtion-cognition-Focused educatIonal programme to reduce diabetes-related distress in Malay adults with Type 2 diabetes. (DOCX 17 kb)

## Abbreviations

AG: Attention-control group
ANOVA: Analysis of variance
BIPQ: Brief Illness Perception Questionnaire
BP: Blood pressure
CONSORT: Consolidated Standards of Reporting Trials
DASS: Depression, Anxiety and Stress Scale
DDS-17: 17-item Diabetes Distress Scale
DFP: Diabetic foot problems
DMSES: Diabetes Management Self Efficacy Scale
DRD: Diabetes-related distress
FMS: Family medicine specialists
HC: Health clinics
HDL-C: High density lipoprotein-cholesterol
ICC: Intraclass correlation coefficient
LDL-C: Low density lipoprotein-cholesterol
M&HO: Medical and health officers
MREC: Medical Research Ethics Committee
PA-CESD: Positive Affects subscale of the Center for Epidemiologic Studies Depression Scale
PHQ-9: 9-item Patient Health Questionnaire
SDSCA: Diabetes Self-Care Activities
T2DM: Type 2 diabetes mellitus
TG: Triglyceride
US: United States of America
VEMOFIT: Value-based emotion-cognition-focused educational programme in adults with T2DM
VG: VEMOFIT group
WHOQOL-BREF: World Health Organization Quality of Life- Brief
