# Supplementary material for: The effectiveness of a value-based EMOtion-cognition-Focused educatIonal programme to reduce diabetes-related distress in Malay adults with Type 2 diabetes (VEMOFIT): study protocol for a cluster randomised controlled trial
Source: BMC Endocr Disord. 2017 Apr 4;17:22. doi: 10.1186/s12902-017-0172-8 (PMC5379686; doi:10.1186/s12902-017-0172-8)
Supplement: Additional file 1: — The timetable. T2DM: type 2 diabetes mellitus; VG: VEMOFIT group; AG: attention-control group. VEMOFIT: Value-based EMOtion-cognition-Focused educatIonal programme to reduce diabetes-related distress in Malay adults with Type 2 diabetes. (DOCX 17 kb) [file 12902_2017_172_MOESM1_ESM.docx]

| **Session**  2-hours per session | **Content** | **Home-work/**  **Evaluation** |
| --- | --- | --- |
| **Session 1 (Week 0)**  (about 2-hour)  10 minutes  10 minutes  10 minutes  15 minutes  40 minutes  10 minutes  15 minutes  10 minutes  (90 minutes) | **Illness perception & T2DM disease education**   - Introduction to the outline of the whole programme - Ice-breaking- members introduction - Personal meanings of diabetes mellitus - **Talk 1a**: the role of illness perceptions & personal health beliefs in diabetes self-care - **Activity 1**: Exploration- reflection on own perceptions, comparing them with the significant other’s ones   Break   - **Talk 1b**: diabetes mellitus- the disease - Summary for the day   Or   - **Discussion 1 (AG)**: on feeling about and coping with T2D | 1. (**VG**) Value exploration and synchronization in the family 2. (**VG** & **AG**) T2DM information leaflet |
| **Session 2 (Week 2)**  5 minutes  30 minutes  10 minutes  15 minutes  40 minutes  10 minutes  10 minutes | **Emotion Skills- understanding emotions**   - Welcome notes - **Discussion 1**: on diabetes-related distress in relation to illness perceptions and other sources of stress   Break   - **Talk 2**: emotions and health - **Activity 2**: understanding emotions & relaxation technique - Relaxation technique - Summary for the day | 1. (**VG**) The VEMOFIT Diary 2. (**VG**) Providing social support leaflet |
| **Session 3 (Week 4)**  5 minutes  30 minutes  10 minutes  5 minutes  40 minutes  10 minutes  10 minutes | **Emotion Skills- emotion management**   - Welcome notes - **Discussion 2**: on emotional skills/experience   Break   - **Talk 3**: Emotions and diabetes mellitus - **Activity 3**: managing emotions - Practising relaxation - Summary for the day | (**VG**) The VEMOFIT Diary |
| **Session 4 (Week 6)**  10 minutes  30 minutes  10 minutes  20 minutes  20 minutes  20 minutes  10 minutes  (90 minutes) | **The value-emotion-cognition framework**   - Welcome notes - **Discussion 3**: on emotion skills/experience   Break   - **Talk 4**: The value-emotion-cognition framework for self-care - **Activity 4**: self-management goal-setting - Post-intervention evaluation- Primary and self-reported secondary outcomes measurement - Summary for the day- remind on the future refresher/evaluations at 3-month and 6-month   Or   - **Discussion 2 (AG)**: on social support at home | 1. (**VG**) The VEMOFIT Diary 2. (**VG** & **AG**) Blood tests within 2 weeks |
| **3-month Booster (week 18)**  5 minutes  30 minutes  10 minutes  30 minutes  5 minutes  10 minutes | **Booster session at 3-month**   - Welcome notes  1. **Discussion 4**: on self-management goals-barriers, on emotional skills/experience   Break   1. **Talk 4 (repeat)**: The value-emotion-cognition framework 2. Evaluation of the programme by the participants 3. Summary for the day | 1. (**VG**) The VEMOFIT Diary      1. (**VG** & **AG**) Blood tests within 2 weeks |
| **6-month and 12-month evaluation**  15 minutes  45 minutes  15 minutes  45 minutes  (90 minutes) | Welcome notes   1. Primary and secondary outcomes measurement   Break   1. **Discussion 4 (repeat)**: on self-management goals- barriers, on emotion skills/experience   Or  **Discussion 3 (AG)**: on treatment and care satisfaction received at the respective clinics | 1. (**VG**) The VEMOFIT Diary 2. (**VG** & **AG**) Blood tests within 2 weeks |
